# Smartphone App Use for Diabetes Management: Evaluating Patient Perspectives

**DOI:** 10.2196/diabetes.6643

**Published:** 2017-01-23

**Authors:** Kirstie Lithgow, Alun Edwards, Doreen Rabi

**Affiliations:** 1 Department of Medicine Cumming School of Medicine University of Calgary Calgary, AB Canada; 2 Institute for Public Health Cumming School of Medicine University of Calgary Calgary, AB Canada

**Keywords:** type 1 diabetes, mobile health, smartphone

## Abstract

**Background:**

Finding novel ways to engage patients in chronic disease management has led to increased interest in the potential of mobile health technologies for the management of diabetes. There is currently a wealth of smartphone apps for diabetes management that are available for free download or purchase. However, the usability and desirability of these apps has not been extensively studied. These are important considerations, as these apps must be accepted by the patient population at a practical level if they are to be utilized.

**Objective:**

The purpose of this study was to gain insight into patient experiences related to the use of smartphone apps for the management of type 1 diabetes.

**Methods:**

Adults with type 1 diabetes who had previously (or currently) used apps to manage their diabetes were eligible to participate. Participants (n=12) completed a questionnaire in which they were required to list the names of preferred apps and indicate which app functions they had used. Participants were given the opportunity to comment on app functions that they perceived to be missing from the current technology. Participants were also asked whether they had previously paid for an app and whether they would be willing to do so.

**Results:**

MyFitnessPal and iBGStar were the apps most commonly listed as the best available on the market. Blood glucose tracking, carbohydrate counting, and activity tracking were the most commonly used features. Ten participants fulfilled all eligibility criteria, and indicated that they had not encountered any one app that included all of the functions that they had used. The ability to synchronize an app with a glucometer or insulin pump was the most common function that participants stated was missing from current app technology. One participant had previously paid for a diabetes-related app and the other 9 participants indicated that they would be willing to pay.

**Conclusions:**

Despite dissatisfaction with the currently available apps, there is interest in using these tools for diabetes management. Adapting existing technology to better meet the needs of this patient population may allow these apps to become more widely utilized.

## Introduction

Finding novel ways to engage patients in chronic disease management has led to an increased interest in the potential of mobile health (mHealth) technologies for the management of diabetes. There is currently a wealth of smartphone apps for diabetes management that are available for free download or purchase [1]. The functions of diabetes apps vary, with glucose tracking, calorie counting, activity tracking, and education among the many available features [2,3]. There is evidence from small studies that app use may have a beneficial effect on health outcomes [4], however user preferences and desired features of these apps is a topic in need of further study [5,6]. A previous systematic review on this topic demonstrated that the usability of many apps is suboptimal, and advocated for greater patient input in the development of such apps [1]. Conway et al [7] illustrated that preferences of diabetes app users may not be well represented in the available technology, as education features are highly desired, yet only present in a minority of apps. There also appear to be differences in user preferences according to gender [8] and age [1,9]. Other studies [9,10] have highlighted the importance of user satisfaction and ease of use in acceptance of apps as diabetes self-management tools. These are important considerations, as these apps must be accepted by the patient population at a practical level if they are to be utilized. The objective of this study was to gain insight into patient perspectives and experiences related to the use of available diabetes apps. Specifically, we were interested in which diabetes apps were being used, what features of these apps were valued by patients, and what perceived gaps exist in app technology.

## Methods

**Figure 1:**
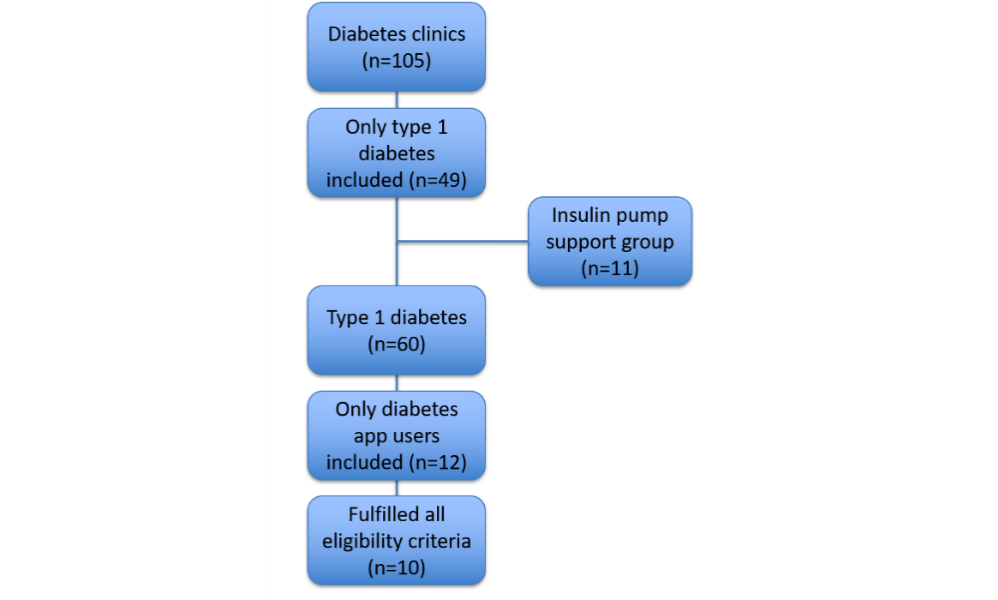
Patient recruitment strategy.

The procedures followed in this study were in accordance with the ethical standards of the Conjoint Health Research Ethics Board. Over a period of 8 months we recruited patients from nine diabetes clinics, one diabetes and pregnancy clinic, and one insulin pump support group to participate in a short survey about their experiences using smartphone apps for diabetes. We used a purposive sampling strategy to target patients with a high probability of having sufficient experience with smartphone apps. Recent work with a patient portal at our center suggested that patients with type 1 diabetes have higher rates of e-literacy and diabetes self-efficacy than patients with type 2 diabetes. Based on this premise, we limited our recruitment to patients with type 1 diabetes. To be eligible for participation, patients had to have a diagnosis of type 1 diabetes mellitus, along with experience using smartphone apps for some aspect of their diabetes management. During recruitment, each clinic schedule was reviewed in advance to identify patients with type 1 diabetes. These individuals were then approached and screened for eligibility based on whether they had any experience using smartphone apps for diabetes self-management. A total of 60 patients were approached for recruitment from the clinics (n=49) and support group (n=11; Figure 1). The high number of patients that did not participate was due to ineligibility based on a lack of experience with smartphone apps; all eligible patients chose to participate. Our original targeted sample size was 20, but recruitment was stopped at 12 patients due to time constraints and the emergence of some consistent patterns.

The 12 eligible participants answered a short questionnaire. Participants were required to provide demographic information including age, gender, number of years with type 1 diabetes diagnosis, number of years using a smartphone, and what type of smartphone they were using. Participants were asked to provide the specific names of up to 3 of the best apps they had encountered for managing their diabetes. From a list of known functions of available diabetes apps, participants were asked to indicate which features they had used. Participants were then asked if any of the apps they had encountered included all of the functions that they had used, and were asked to provide the names of any such apps. Participants were given space to make specific comments about app functions that they perceived were absent from currently available technology. Finally, participants were asked whether they had ever paid for an app, along with if (and how much) they would be willing to pay.

## Results

**Figure 2:**
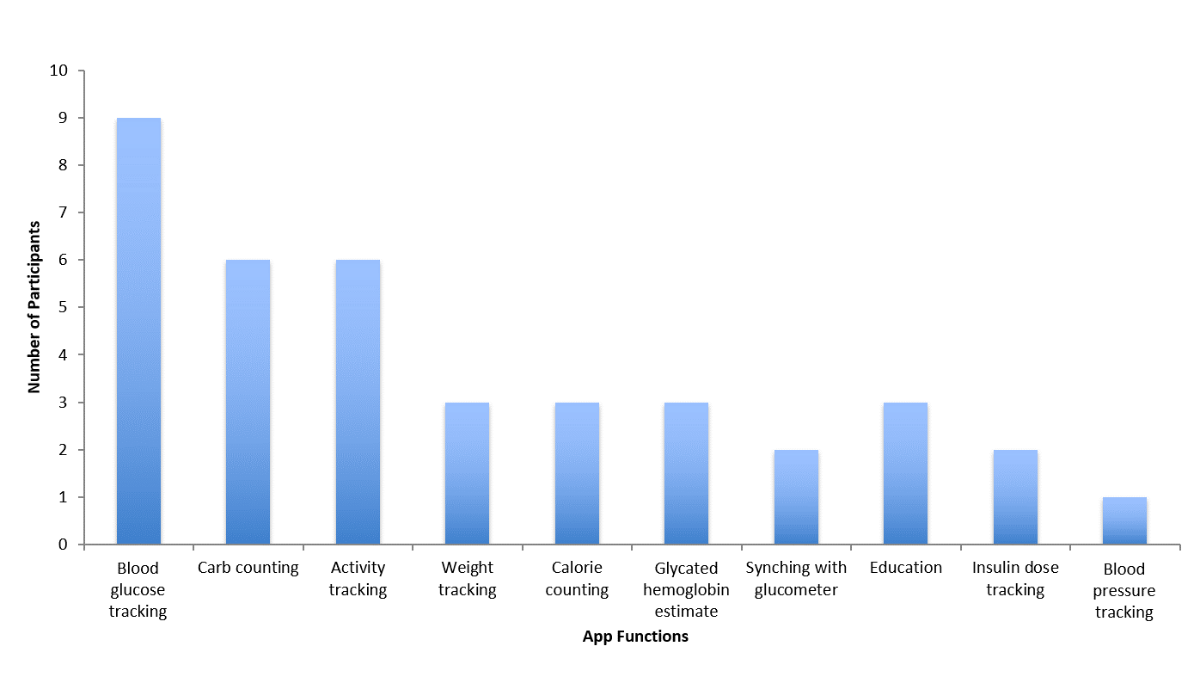
Use of different app functions, as self-reported by participants.

Data was collected from 12 eligible participants, however 2 respondents were excluded from the final analysis, as incomplete data was provided. The age of participants ranged from 18 to 64 years (mean 34.5). We recruited 7 men and 3 women. The average length of time with a diagnosis of type 1 diabetes was 20.6 years. We did not collect data regarding the use of insulin pumps or continuous glucose monitors (CGMs). MyFitnessPal and iBGStar were the apps most commonly listed as the best available on the market; each was listed by 2 participants. The remainder of the apps that were listed included Diabetes App, Guide Resto, Glucose Buddy, Carb Control, Lose it, S health, Bike tracks, Mountain Bike Pro, and iPhone health App; each of these apps was listed by 1 participant. Two participants indicated that they were no longer using apps due to dissatisfaction with apps that they had previously used. In terms of functions used, blood glucose tracking, carbohydrate counting, and activity tracking were the most commonly used features (Figure 2). In response to whether any apps they had encountered included all functions that they were using, all participants answered *no*. The function most frequently indicated by participants as being absent from app technology was the ability to synch the app with a glucometer or insulin pump, which was a response given by 5 participants. *Reminder to check blood glucose* was indicated as a missing feature by 2 participants, *Canadian units for blood glucose values* was mentioned by 1 participant, and a *more comprehensive graphing function* was listed by 1 participant. Only 1 participant reported previously paying for an app related to diabetes management, and 9 patients indicated that they would be willing to pay for an app. Of the participants willing to pay, 8 stated that they would be willing to pay between Can $5 and $20, and 1 participant indicated that there was no upper limit to what they would be willing to pay.

## Discussion

Despite the presence of hundreds of diabetes-related apps, recruiting patients that currently use these apps was a challenge. Furthermore, the apps that are currently being used do not meet all of the patients’ expectations of a self-management tool. However, there remains a desire to continue using these apps despite their shortcomings, and a high willingness to pay for this technology.

We acknowledge that this study has some limitations regarding its generalizability. The collected data represent the views of a population of patients with type 1 diabetes, which may differ from patients with type 2 diabetes. These findings do not extend to mobile apps that might be part of a larger patient or electronic medical record platform. In our small study, we were not able to make any specific correlations with the nature of app use and patient factors, such as duration of diabetes, gender, and concurrent use of insulin pump or CGM. Previous studies have shown that duration of diabetes diagnosis influences how patients engage with self-management technology [11]. An average duration of 20.6 years of diabetes diagnosis reflects extensive experience with diabetes self-management, which may limit the perceived value of diabetes apps by our participants.

The most commonly used functions cited by participants (blood glucose tracking, carbohydrate counting, and activity tracking) all relate to logging and tracking data, while more sophisticated functions such as education, feedback, and social networking were relatively underutilized. Further analysis of the specific apps preferred by participants revealed that the functions performed by these apps align with the most commonly used functions. This finding may suggest that the nature of participant app use is primarily based on what functions are included in currently available apps, and does not necessarily reflect patient preferences. Previous authors have illustrated that many patients feel that existing apps are missing important functions [9]. From a patient perspective, education has been specifically identified as an area in need of further development for diabetes apps [7]. Importantly, perceived lack of additional benefit from apps for diabetes management has been shown to be a barrier to app use [9]. Development of apps that take patients’ desires and preferences into consideration, and offer novel tools for self-management, is therefore necessary to improve patient engagement.

Interestingly, despite the ubiquitous use of smartphones and high acceptance of mHealth by patients, only a small proportion of patients are currently utilizing smartphone technology to manage their diabetes [7]. One factor potentially contributing to this trend is that most currently available apps are not capable of synchronization with a glucometer or insulin pump.

One insulin pump user specifically commented that carrying both an insulin pump and a smartphone while doing more intense physical activity was unappealing. Lack of interoperability of apps with other devices has been identified as an area of concern from a patient perspective [9]. Redundant data tracking and entry into a smartphone app, in addition to an insulin pump or glucometer, could therefore be an important barrier to utilization of diabetes apps by patients, representing a potential implication for future development in mHealth technology.

Finally, while the vast majority of participants had never paid for an app, an equal proportion expressed a willingness to do so. Although the overall results of this study reflect general dissatisfaction with the currently available technology, these findings suggest that there is desire and interest for using diabetes apps from a patient perspective. To ensure that these tools are fully harnessed, existing technology must be adapted to better meet the needs of this patient population.

## Abbreviations

CGM: continuous glucose monitor
mHealth: mobile health

## References

[1] Arnhold M, Quade M, Kirch W (2014). Mobile applications for diabetics: a systematic review and expert-based usability evaluation considering the special requirements of diabetes patients age 50 years or older. J Med Internet Res.

[2] Tran J, Tran R, White JR (2012). Smartphone-based glucose monitors and applications in the management of diabetes: an overview of 10 salient “apps” and a novel smartphone-connected blood glucose monitor. Clinical Diabetes.

[3] Rao A, Hou P, Golnik T, Flaherty J, Vu S (2010). Evolution of data management tools for managing self-monitoring of blood glucose results: a survey of iPhone applications. J Diabetes Sci Technol.

[4] Garabedian LF, Ross-Degnan D, Wharam JF (2015). Mobile phone and smartphone technologies for diabetes care and self-management. Curr Diab Rep.

[5] Chomutare T, Fernandez-Luque L, Arsand E, Hartvigsen G (2011). Features of mobile diabetes applications: review of the literature and analysis of current applications compared against evidence-based guidelines. J Med Internet Res.

[6] Holtz B, Lauckner C (2012). Diabetes management via mobile phones: a systematic review. Telemed J E Health.

[7] Conway N, Campbell I, Forbes P, Cunningham S, Wake D (2016). mHealth applications for diabetes: user preference and implications for app development. Health Informatics J.

[8] Arora S, Ford K, Terp S, Abramson T, Ruiz R, Camilon M, Coyne CJ, Lam CN, Menchine M, Burner E (2016). Describing the evolution of mobile technology usage for Latino patients and comparing findings to national mHealth estimates. J Am Med Inform Assoc.

[9] Scheibe M, Reichelt J, Bellmann M, Kirch W (2015). Acceptance factors of mobile apps for diabetes by patients aged 50 or older: a qualitative study. Med 2 0.

[10] Kim YJ, Rhee SY, Byun JK, Park SY, Hong SM, Chin SO, Chon S, Oh S, Woo J, Kim SW, Kim YS (2015). A smartphone application significantly improved diabetes self-care activities with high user satisfaction. Diabetes Metab J.

[11] Klasnja P, Kendall L, Pratt W, Blondon K (2015). Long-term engagement with health-management technology: a dynamic process in diabetes. AMIA Annu Symp Proc.

